# Garlic Attenuates Cardiac Oxidative Stress via Activation of PI3K/AKT/Nrf2-Keap1 Pathway in Fructose-Fed Diabetic Rat

**DOI:** 10.1371/journal.pone.0094228

**Published:** 2014-05-05

**Authors:** Raju Padiya, Debabrata Chowdhury, Roshan Borkar, R. Srinivas, Manika Pal Bhadra, Sanjay K. Banerjee

**Affiliations:** 1 Division of Medicinal Chemistry and Pharmacology, Indian Institute of Chemical Technology, Hyderabad, India; 2 Division of Chemical Biology, Indian Institute of Chemical Technology, Hyderabad, India; 3 National Centre for Mass Spectrometry, Indian Institute of Chemical Technology, Hyderabad, India; University of Western Ontario, Canada

## Abstract

**Background:**

Cardiovascular complication due to diabetes has remained a major cause of death. There is an urgent need to intervene the cardiac complications in diabetes by nutritional or pharmacological agents. Thus the present study was designed to find out the effectiveness of garlic on cardiac complications in insulin-resistant diabetic rats.

**Methods and Results:**

SD rats were fed high fructose (65%) diet alone or along with raw garlic homogenate (250 mg/kg/day) or nutrient-matched (65% corn starch) control diet for 8 weeks. Fructose-fed diabetic rats showed cardiac hypertrophy, increased NFkB activity and increased oxidative stress. Administration of garlic significantly decreased (p<0.05) cardiac hypertrophy, NFkB activity and oxidative stress. Although we did not observe any changes in myocardial catalase, GSH and GPx in diabetic heart, garlic administration showed significant (p<0.05) increase in all three antioxidant/enzymes levels. Increased endogenous antioxidant enzymes and gene expression in garlic treated diabetic heart are associated with higher protein expression of Nrf2. Increased myocardial H_2_S levels, activation of PI3K/Akt pathway and decreased Keap levels in fructose-fed heart after garlic administration might be responsible for higher Nrf2 levels.

**Conclusion:**

Our study demonstrates that raw garlic homogenate is effective in reducing cardiac hypertrophy and fructose-induced myocardial oxidative stress through PI3K/AKT/Nrf2-Keap1 dependent pathway.

## Introduction

Type 2 diabetes mellitus is a major lifestyle disorder of the 21^st^century and associated with cardiac complications. Coronary artery diseases and hypertension are leading cause of death in diabetic patients. However, along with micro- and macro-vascular complication, heart failure due to cardiac dysfunction is very common with diabetes [1]. The pathogenesis of cardiac dysfunction associated with diabetes is due to insulin resistance and changes in different metabolic parameters. It is a big challenge for the researchers to investigate the molecular defects underling the cardiac changes and to find a way to prevent it,

High blood levels of insulin, free fatty acid and glucose as well as oxidative stress can alter important signalling pathways and may increase cardiovascular risk [2]. In our previous study we confirmed that high-fructose diet leads to insulin resistance and increased hepatic oxidative stress [3]. Metabolic complication and insulin resistance in fructose-fed rats can lead to cardiac complications such as oxidative stress and hypertrophy, and alter cellular structure and function. Cardiac hypertrophy and oxidative stress has been observed previously in response to fructose feeding [4]. A causal role for oxidative stress in the development of cardiovascular complications in diabetes is increasingly recognized [5]. Similarly, a specific link between oxidative stress and insulin resistance is also well established [6].

Despite the availability of anti-diabetic drugs for the management of diabetes, cardiovascular complication is the leading cause of death in diabetic patients. Thus there is an urgent need to intervene the cardiac complications by nutritional or pharmacological agents. The anti-diabetic effect of garlic is well established in different animal models of type I as well as type II diabetes [7]. Despite the promising evidence of effectiveness of raw garlic in reducing hyperglycemia, little is known about its effectiveness for ameliorating cardiac complications in insulin-resistance diabetic animals.

Thus the present study was designed to investigate whether raw garlic can attenuate pathological changes on fructose fed type 2 diabetic heart and to find the molecular signalling pathway responsible for this beneficial effect.

## Materials and Methods

### Chemicals and antibodies

Garlic was purchased from local market Hyderabad, India. Antibodies like Anti-GAPDH, (IMG6665A) is from Imgenes, Anti-NFkB p50 (251563), Anti-NFkB p65 (C22B4) and Anti-Nrf2 (251445) are from Abbiotec, Anti- AKT (9272S), phospho-Akt (9271S), Anti-PI3K class III (D4E2) (3358S), Keap1 (8047S) are from Cell Signaling, phospho-PI3K (3332R) from Bioss and Anti-Lamin A MAB3540 from Millipore, Anti-Rabbit IgG-HRP and Anti-mouse IgG-HRP are from Santa Cruz. All other chemicals were obtained from Sigma, USA.

### Preparation of garlic Homogenate

Garlic was purchased from local market Hyderabad, India. Individual bulbs were put in a grinder to form a juicy paste as described. The garlic homogenate was prepared freshly each day.

### Determination of garlic constituent by LC/MS

The supernatant of garlic homogenate were filtered through 0.45 µm glass filter and diluted further to make appropriate solution. 20 µL was injected in LC-MS. The analysis was carried out on an Agilent 1200 series HPLC instrument (Agilent Technologies, USA) equipped with a quaternary pump (G13311A, USA), a de-gasser (G1322A, USA), a diode-array detector (G1315D, USA), an auto sampler (G1329A, USA) and a column compartment (G1316A, USA). Mass spectrometric detection was carried out on a quadrupole time-of-flight (Q-TOF) mass spectrometer (Q-TOF LC/MS 6510 series classic G6510A, Agilent Technologies, USA) equipped with an ESI source. The data acquisition was under the control of Mass Hunter workstation software. The typical operating source conditions for MS scan in positive ion ESI mode were optimized as follows; the fragmentor voltage was set at, 80 V; the capillary at, 3000–3500 V; the skimmer at, 60 V; nitrogen was used as the drying (300°C; 9 L/min) and nebulizing (45 psi) gas. For full scan MS mode, the mass range was set at *m/z* 100–2000. All the spectra were recorded under identical experimental conditions and are average of 20–25 scans.

### Animals and Treatment

All animal experiments were undertaken with the approval of Institutional Animal Ethical Committee of Indian Institute of Chemical Technology (IICT), Hyderabad (Approval no. IICT/PHARM/SKB/11/08/10). Male Sprague-Dawley rats (200–250 gms) were purchased from the National Institute of Nutrition (NIN), Hyderabad, India. The animals were housed in BIOSAFE, an animal quarantine facility of the Indian Institute of Chemical Technology (IICT), Hyderabad, India. The animal house is maintained at temperature 22±2°C with relative humidity 50±15% and 12 hour dark/light cycle throughout the study. Rats were randomly divided into three groups (n = 7). Control group (Control) was fed 65% corn starch diet (Research diet, USA), whereas diabetic group (Diabetic) was fed 65% fructose diet (Research diet, USA) for the induction of diabetes and associated metabolic [3]. The third group (Dia+Garl) was fed 65% fructose diet along with raw garlic homogenate (250 mg/kg) for a period of 8 weeks.

### Sample collection and biochemical assay

The animals of all three groups were sacrificed after 8 weeks of study. Cardiac tissues were collected and stored at −80°C for further biochemical evaluation. Each heart tissue was homogenized with 20 times volume of heart weight in ice cold 0.05 M potassium phosphate buffer (pH-7.4) and treated separately for different measurements.

### Estimation of heart weight/body weight ratio

In each group, heart weight/body weight ratio was measured on the day of sacrifice. Heart weight was measured after keeping the heart in ice cold saline and squeezing out the blood.

### TBARS, Conjugated dienes and Nitric oxide

The extent of lipid peroxidation in hearts was determined by measuring malondialdehyde (MDA), conjugated dienes and nitric oxide. Malondialdehyde (MDA) content was determined according to modified method based on the reaction with thiobarbituric acid [8]. Data were expressed as nanomoles per gm heart weight using extinction co-efficient of 1.56×10^−5^ M^−1^ cm^−1^. Conjugated dienes was also measured as a marker of lipid peroxidation by a biochemical method [8]. Nitric oxide, another marker of oxidative stress, was determined by a commercially available kit (Assay design, USA). Assay is based on reduction of NO_3_^−^ into NO_2_^−^ using nitrate reductase. The azo dye is produced by diazotization of sulfanilic acid (Griss Reagent-1) with NO_2_^−^ and then subsequent coupling with N-(1-napthyl)-ethylene diamine (Griss Reagent-2). The azo dye was measured calorimetrically at 540 nm. NO level was expressed as μmol/gm heart.

### Measurement of ROS

Reactive oxygen species (ROS) was measured fluorometrically in heart tissue homogenates using 2,7-dichlorofluorescein diacetate (DCF-DA). Briefly 100 µM of DCF-DA and tissue homogenate was incubated for 30 min at room temperature in dark. After incubation, the volume of the reaction was adjusted with phosphate buffer saline (PBS, 0.1 M, pH-7.4) and fluorescence was measured at 488 nm excitation and 525 nm emission wavelength. The data obtained was expressed as percentage of control [9].

### NFkB Activity

The cardiac NFkB activity was determined by the kit obtained from Cayman Chemical (Cat. 10007889). This is a non-radioactive and sensitive method for detecting DNA-binding activity of NFkB obtained from tissue extracts.

### Catalase, SOD, GSH and GPx

Catalase, GSH and GPx were measured by biochemical methods as described [10]. SOD activity in the myocardial homogenate was determined according to the standard assay kit (Fluka Analytical, Switzerland, Catalog No. 19160).

### Hydrogen Sulphide (H_2_S) and Protein estimation

H_2_S concentration was measured as described earlier [3]. Briefly, 0.1 ml supernatant was added into a test tube containing 0.125 ml 1% zinc acetate and 0.15 ml distilled water. Then 0.067 ml 20 mM N, N-dimethyl-phenylene diamine dihydro chloride in 7.2M HCL was added. This was followed by addition of 0.067 ml 30 mM FeCl_3_ in 1.2 M HCL. The absorbance of resulting solution was measured with a spectrophotometer at a wavelength of 670 nm. The H_2_S concentration in a solution was calculated according to the calibration curve of sodium hydrogen sulphide (NaHS: 3.12–400 µmol) and data were expressed as H_2_S concentration in μmol/gm heart. Protein concentrations were measured from heart supernatant [3].

### Gene expression

Total RNA was extracted from a piece of 0.2 g heart with Trizol (Sigma). Gene expression of ANP, β-MHC, Nrf2, KEAP and MnSOD were performed [8]. Briefly, cDNA was prepared from RNA and PCR was performed in a 0.2 ml tube containing 2 µl cDNA, 1 µl of (10 pmol) each forward and reverse primers, 2.0 µl of dNTP (1.25 mM each nucleotide), 2.5 µl of 10× PCR Buffer, 0.25 µl of Taq polymerase and 11.25 µl of dH_2_0. After denaturizing at 95°C for 5 min, the whole mixture underwent PCR at 95°C for 60 seconds, 61°C (annealing temperature) for 60 seconds, 72°C for 60 seconds for 32 cycles. The PCR products were separated on 2% agarose gel stained with ethidium bromide. The optical density of bands was measured by using the Gel Documentation System. The analysis was based on reference gene RPL32. The sequence of the primers are summarised in Table 1.

**Table 1:** List of primer sequences for RT-PCR.

Gene	Primers	Sequence
**ANP**	Forward	5-GAGAAGATGCCGGTAGAAGA-3
	Reverse	5-AGAGCACCTCCATCTCTCTG-3
**β-MHC**	Forward	5-TGGAGCTGATGCACCTGTAG-3
	Reverse	5-ACTTCGTCTCATTGGGGATG-3
**Nrf2**	Forward	5-CAGTCTTCACCACCCCTGAT-3
	Reverse	5-GGTAGTCTCAGCCTGCTGCT-3
**MnSOD**	Forward	5-GAACCACAGGCCTTATTCCA-3
	Reverse	5-GGGCTTCACTTCACTTCTTGCAAAC-3
**RPL32**	Forward	5-AGATTCAAGGGCCAGATCCT-3
	Reverse	5-CGATGGCTTTTCGGTTCTTA-3

### Immunoblotting analysis

Separation of nuclear and cytoplasmic fraction was done by using Nuclear and Cytoplasmic Extraction Kit (Thermo Scientific) and whole heart protein for immunoblotting was prepared as described by Banerjee et al., 2010 [11]. Briefly, quantity of protein was measured by Bradford method. An equal amount (50 µg) of protein was separated by sodium dodecyl sulfate polyacrylamide gel electrophoresis (SDS-PAGE). After electrophoresis, protein was transferred to PVDF membranes (Amersham Biosciences). The membranes were then blocked by 5% non-fat dry milk in Tris-buffered saline Tween-20 (TBS-T; 10 mM Tris, pH 7.5, 150 Mm NaCl, 0.05% Tween-20) for 1 h, and subsequently washed and incubated with primary antibody p50NFkB and p65NFkB (1∶1000 dilution, Abbiotec) in TBS-T and 5% non-fat dry milk at 4°C overnight. After washing with TBS-T, membranes were incubated with anti-rabbit horseradish peroxidase conjugated secondary antibody (1∶1500 dilutions, Amersham) for 1 h. Signal was detected by chemiluminescence using the ECL detection system (Amersham). The same procedure was repeated for P-PI3K (1∶1000, Bioss), PI3K (1∶1000, Cell signalling), P-AKT (1∶1000, Cell Signaling), AKT (1∶1000, Cell Signalling), Nrf2 (1∶300 dilution, Abbiotec), Keap1 (1∶1000, Cell Signaling), Lamin A 1∶500 (Millipore) and GAPDH (1∶1000, Imgenes). Gel staining with coomassie blue was used as an internal control for equal loading of protein. Quantification of bands was performed using Image J Software (NIH).

### Statistical analysis

All values are expressed as mean ± SEM. Data were statistically analysed using one way ANOVA for multiple group comparison, followed by student unpaired ‘t’ test for group wise comparison. Significance was set at P<0.05.

## Results

### Determination of allicin and other garlic constituent by LC/MS

**Figure 1:**
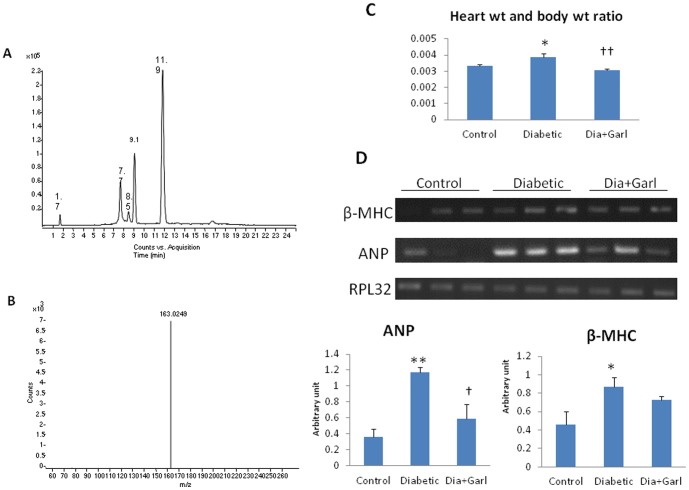
(A) LC-ESI-MS total ion chromatogram (TIC) of garlic extract (B) positive ion ESI-MS spectra of Allicin (*m/z* 163, Rt = 11.9 min). (C) Effect of garlic on heart weight body weight ratio (N = 7) and (D) β-MHC and ANP levels. (N = 3) *p<0.05, **p<0.01 vs. Control group; †p<0.05, ††p<0.01 vs. Diabetic group.

The separated components of garlic are γ-glutamyl-S-allyl-L-cysteine (Rt1.7 min, 291 *m/z*); Alliin (Rt 7.7 min, 178 *m/z*); S-Allyl-l-cysteine, deoxyalliin (Rt 8.5 min, 162 *m/z*); Vinyldithiin (Rt 9.1 min, 145 *m/z*); Allicin (Rt 11.9 min, 163 *m/z*) (Fig. 1A). The total ion chromatogram (TIC) and MS/MS spectrum of protonated allicin are given in Figs. 1A and 1B respectively.

### Cardiac Hypertrophy

A significant (p<0.05) increase in heart/body weight ratio as well as β-MHC and ANP gene expression was observed in diabetic group compared to control group. However, significant (p<0.05) decrease in heart/body weight ratio and ANP gene expression was observed in garlic treated (Dia+Garl) group compared to diabetic group (Fig.1C & 1D).

### TBARS, Conjugated dienes and Nitric Oxide

**Figure 2:**
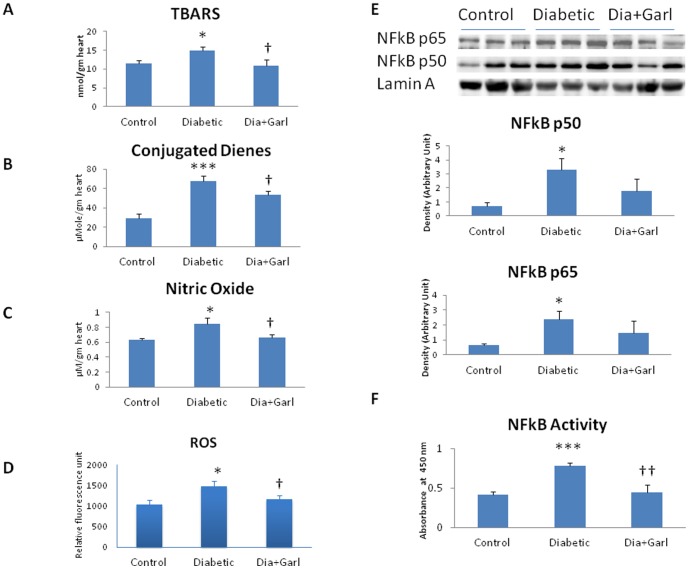
Effect of garlic on (A) TBARS levels, (B) Conjugated diens levels, (C) Nitric oxide levels (D) ROS generation (E) Nuclear NFkB levels after immunoblotting (N = 3) and (F) NFkB activity. (N = 7) *p<0.05, ***p<0.001 vs. Control group; †p<0.05, ††p<0.01 vs. Diabetic group.

A significant (p<0.05) increase in TBARS, conjugated dienes and nitric oxide levels was observed in diabetic group in comparison to control group. However, significant (p<0.05) decrease in all three oxidative stress parameters was observed in garlic treated diabetic (Dia+Garl) group (Fig.2A, 2B, 2C).

### ROS Generation

A significant (p<0.05) increase in ROS levels was observed in diabetic group in comparison to control group. However, significant (p<0.05) decrease in ROS levels was observed in garlic treated diabetic (Dia+Garl) group (Fig.2D).

### Protein expression and activity of NFkB

A significant (p<0.05) increase in p50 and p65 subunit of NFkB levels was observed in diabetic group in comparison to control group. Although, decrease in NFkB levels was observed in garlic treated diabetic (Dia+Garl) group compared to diabetic group, it was not significant (Fig.2E). However, enhanced NFkB activity that observed in diabetic heart was decreased significantly (p<0.05) in garlic treated diabetic (Dia+Garl) group (Fig. 2F).

### Catalase, SOD, GSH, GPx and H_2_S

**Figure 3:**
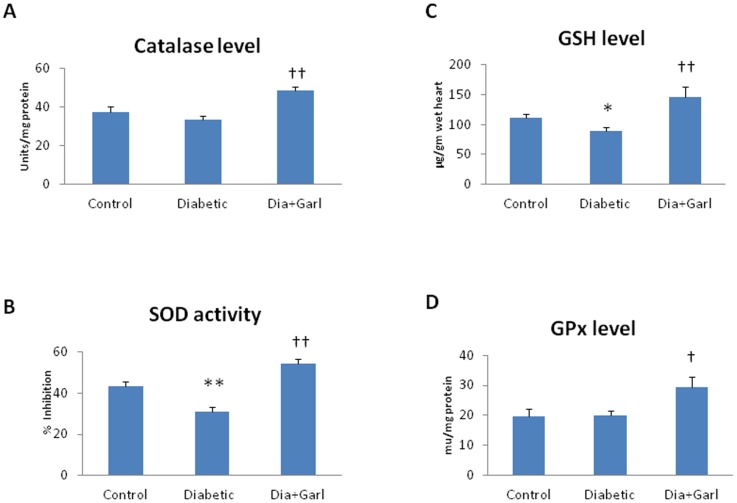
Effect of garlic on myocardial (A) Catalase activity, (B) SOD activity, (C) GSH levels and (D) GPx activity. (N = 7) *p<0.05, **p<0.01 vs. Control group; †p<0.05, ††p<0.01 vs. Diabetic group.

There was no change in myocardial catalase and GPx activity in diabetic group compared to control group. However, a significant (p<0.05) increase in myocardial catalase and GPx activity was observed in garlic treated diabetic (Dia+Garl) group compared to diabetic group (Fig. 3A & 3D).

A significant (p<0.05) decrease in myocardial SOD activity, GSH and H_2_S levels was observed in diabetic group compared to control group. However, there was significant (p<0.01) increase in all parameters observed in garlic treated diabetic (Dia+Garl) group compared to diabetic group (Fig. 3B, 3C & 4A).

**Figure 4:**
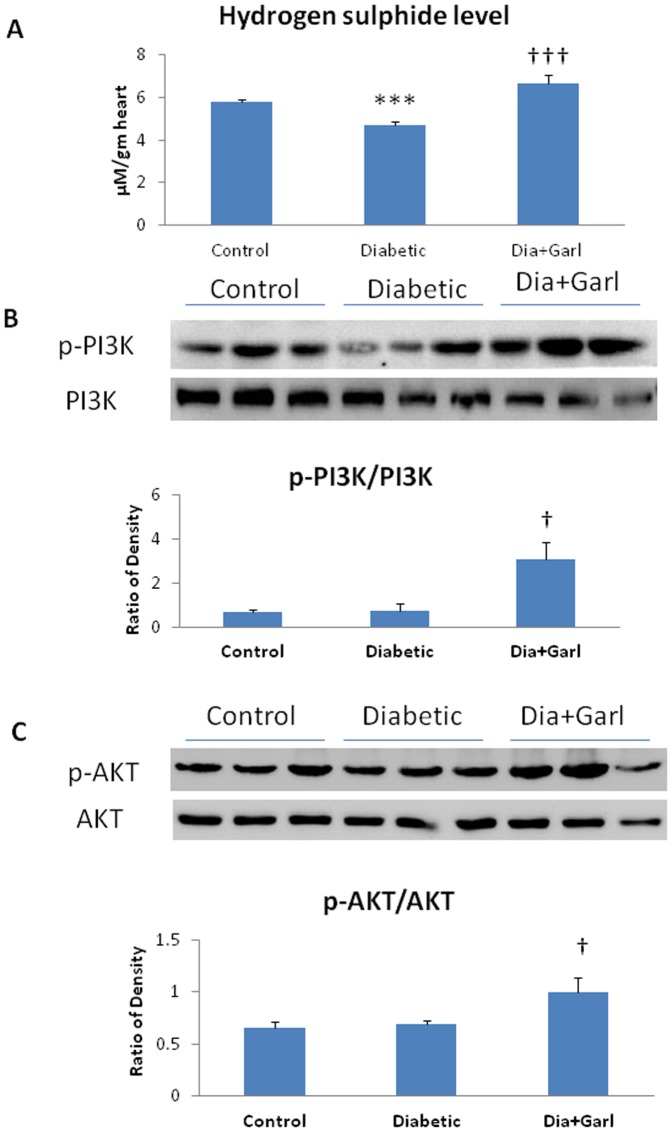
Effect of garlic on (A) myocardial H_2_S levels (N = 7), (B) Immunoblotting of p-PI3K and PI3K, and their ratio (N = 3) (C) immunoblotting of p-AKT and total AKT, and their ratio (N = 3). ***p<0.001 vs. Control group; †p<0.05, †††p<0.001 vs. Diabetic group.

### Gene expression

**Figure 5:**
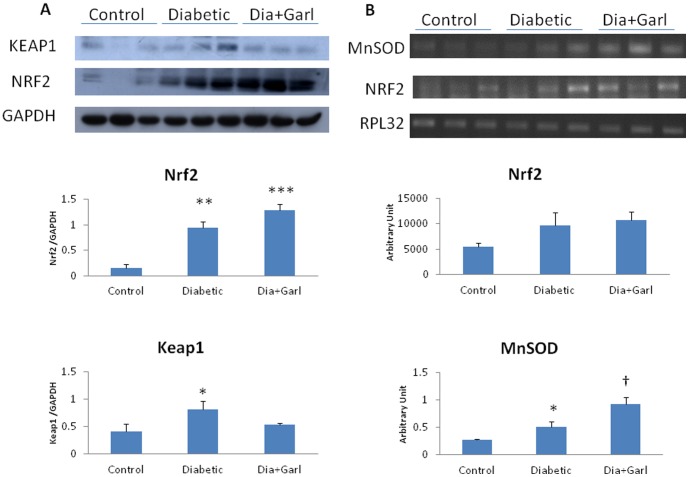
Effect of garlic on (A) immunoblotting of Nrf2 and Keap1, and (B) gene expression of Nrf2 and MnSOD. (N = 3) *p<0.05, **p<0.01, ***p<0.001 vs. Control group; †p<0.05 vs. Diabetic group.

Although an increase in Nrf2 expression was observed in both diabetic and garlic treated diabetic (Dia+Garl) group in comparison to control group but it was not significant. A significant (p<0.05) increase in MnSOD expression was observed in diabetic group compared to control group. Further increase (p<0.05) in MnSOD expression was observed in garlic treated diabetic (Dia+Garl) group compared to diabetic group (Fig. 5B).

### Immunoblotting

Although the ratio of p-PI3K/PI3K and p-AKT/AKT were not changed in diabetic group compared to control group, significant (p<0.05) increase in both ratio was observed in garlic treated (Dia+Garl) group compared to diabetic group (Fig. 4B & 4C). Nrf2 protein levels were significantly (p<0.01) increased in both diabetic and garlic treated (Dia+Garl) groups compared to control group (Fig. 5A). Keap1 protein levels were increased (p<0.05) in diabetic group compared to control group and decreased in garlic treated diabetic (Dia+Garl) group compared to diabetic group (Fig. 5A).

## Discussion

High fructose-induced insulin resistance is associated with an overproduction of superoxide anion in aorta and heart, and promotes cardiovascular alterations such as hypertension, vascular disorder and cardiac hypertrophy [12]. Vascular and cardiac hypertrophy was associated with ROS generation at different times after the initiation of fructose-enriched diet [13]. We observed significant increase in heart weight and body weight ratio, and higher expression of ANP and β-MHC in fructose fed diabetic rat heart. However, administration of raw garlic (rich with allicin) in fructose-fed rats normalised the cardiac hypertrophy along with hypertrophic gene expression. To confirm the presence of allicin we have done LC-MS study. Our data indicated that the raw garlic homogenate that used for the present study was rich with allicin and other compounds like γ-glutamyl-S-allyl-L-cysteine, Alliin, S-Allyl-l-cysteine, deoxyalliin and Vinyldithiin. Previously, allicin, γ-glutamyl-S-allyl-L-cysteine and S-allyl cysteine were reported to have antioxidant property by direct or indirect effect [14]. However, the molecular mechanism behind the beneficial/antioxidant effect of raw garlic was not investigated in heart.

In the present study, high fructose feeding increased oxidative stress as evidenced by elevation of myocardial conjugated dienes, TBARS and cardiac nitric oxide levels, and reduced the myocardial endogenous antioxidants like GSH and SOD. Increased myocardial oxidative stress in fructose-fed rats may also responsible for increased NFkB activity. Increased activity of NFkB provides a molecular mechanism responsible for inflammation and insulin resistance in type 2 diabetes mellitus under basal conditions [15]. Similarly, we also observed increased NFkB activity along with significant increase in nuclear p50 and p65 NFkB protein in diabetic heart. However, administration of raw garlic extract reduced the NFkB activity as well as p50 and p65 NFkB protein levels in diabetic heart.

The present study showed that freshly prepared homogenate of garlic increased H_2_S levels and reverses cardiac pathological changes in fructose-fed insulin resistance rats. Previously, it has been shown that fresh raw garlic homogenate generates H_2_S after interaction with in cellular proteins [16]. It has already been reported that H_2_S scavenge ROS directly, and activates endogenous antioxidant defences [17]. H_2_S reduced oxidative stress in the heart through Nrf2-dependent pathway [18]. Nrf2 is a transcription factors, modulates the gene expression of a number of enzymes including Mn-superoxide dismutase (Mn-SOD) and GPx that serve to detoxify pro-oxidative stressors [18]. Our present study showed that garlic administration increased myocardial Nrf2 expression in fructose fed rat. Increased Nrf2 was also associated with increased myocardial MnSOD expression. Along with Mn-SOD gene expression, garlic administration significantly increased myocardial GSH levels and myocardial SOD, catalase and GPx activity. We believe that increased Nrf2 levels along with endogenous antioxidant after garlic treatment in fructose-fed rats might be responsible for reduction of oxidative stress and cardiac hypertrophy. Although, Nrf2 expression was slightly increased in fructose-fed heart, there was no increase in myocardial Mn-SOD expression as well as endogenous antioxidant activities. Previously it was reported that mild injury or stress can augment Nrf2 levels without increasing antioxidant status [19]. Thus increased expression of Nrf2 during diabetes or oxidative stress as reported previously and in the present study is an adaptive change to overcome cellular damage at early stages of cellular injury.

In the present study, we looked more details regarding the translocation mechanism of Nrf2. In response to oxidative stress, Nrf2 easily dissociates from its repressor protein-Keap, translocates into the nucleus, binds to antioxidant response elements and transactivates the genes of detoxifying and antioxidant enzymes [20]. Significant increase in keap1 expression was observed in fructose fed rat heart. This indicates that Nrf2 was more associated with Keap1 and thus less translocation of Nrf2 in the nucleus to activate antioxidant gene expression. However, administration of raw garlic decreased the keap1 protein expression along with higher Nrf2 expression. Both effects together may increase the nuclear translocation of Nrf2, enhance the expression of antioxidant gene (MnSOD) and increase the endogenous antioxidant activities.

Previously, garlic showed neuroprotective effect by activating the phosphatidylinositol 3-kinase–dependent pathway (PI3K/AKT) [21]. The phosphatidylinositol 3-kinase (PI3K)/AKT pathway plays a major role in cell survival and also required for Nrf2 activation [22]. The present study showed that administration of garlic homogenate in fructose fed rats significantly increased the phospho-PI3K and phospho-AKT protein levels. Increased ratio of p-AKT/AKT might phosphorylate Nrf2 to activate its antioxidant activity and protect heart from oxidative stress.

In conclusion, our study showed that administration of raw garlic homogenate in insulin resistance fructose fed rat activated myocardial Nrf2 through H_2_S and PI3K/AKT pathway, and attenuated cardiac hypertrophy and oxidative stress through augmentation of antioxidant defense system.
